# Outcome measures of the 6 minute walk test: relationships with physiologic and computed tomography findings in patients with sarcoidosis

**DOI:** 10.1186/1471-2466-10-42

**Published:** 2010-08-09

**Authors:** Esam H Alhamad, Shaffi Ahmad Shaik, Majdy M Idrees, Mohammed O Alanezi, Arthur C Isnani

**Affiliations:** 1 Pulmonary Davison, Department of Medicine, King Khalid University Hospital, King Saud University, Riyadh, Saudi Arabia; 2 Department of Family & Community Medicine, King Khalid University Hospital, King Saud University, Riyadh, Saudi Arabia; 3 Pulmonary Medicine, Department of Medicine, Riyadh Military Hospital, Riyadh, Saudi Arabia; 4 Pulmonary Medicine, King Abdulaziz Medical City, Riyadh, Saudi Arabia; 5 College of Medicine and Research Center, King Khalid University Hospital, King Saud University, Riyadh, Saudi Arabia

## Abstract

**Background:**

We assessed the relationship between physiologic parameters, computed tomography patterns, 6 minute walk distance (6MWD) and the distance-saturation product [DSP; defined as the product of the 6MWD and the lowest oxygen saturation during the 6 minute walk test (6MWT)]. In addition, we investigated factors affecting 6MWD in patients with pulmonary sarcoidosis.

**Methods:**

We performed a retrospective study of patient demographics, treatment, pulmonary function, 6MWT, echocardiography and computed tomography results.

**Results:**

Fifty nine patients were included in this study. Their mean+standard deviation age was 47.5 years + 12.5 years, and 42 (71.2%) were female. Mean pulmonary function parameters for forced vital capacity (FVC), forced expiratory volume in 1 second (FEV_1_) and total lung capacity (TLC) results, as percentages of predicted values, were 77.6 ± 22.2, 77.1 ± 22.8 and 78.7 ± 16.1, respectively. Comparison of the DSP with distance walked revealed a significant correlation with factors underlying reduced 6MWD, including gender, pulmonary function indices, partial pressure of oxygen (PaO_2_), and Borg dyspnea score. Other factors were significantly associated with DSP but not distance; these included lung fibrosis (p = 0.02), pulmonary hypertension (p = 0.01) and systemic therapy (p = 0.04). Backward elimination stepwise multiple regression analysis revealed that gender, and FEV_1 _were independent predictors of 6MWD, but FEV_1 _was more strongly related when DSP applied [DSP, R^2 ^= 0.53, p = 0.02; distance, R^2 ^= 0.45, p < 0.0001].

**Conclusion:**

Our findings reveal that, compared to 6MWD alone, the DSP is correlated with a greater number of factors associated with reduced 6MWT performance. Therefore, the DSP may be a useful indicator of functional status in patients with sarcoidosis. Additional large-scale studies are warranted to validate our findings.

## Background

Sarcoidosis is a multisystem disorder characterized by noncaseating granulomas that most commonly affect the lungs and lymph nodes. Despite extensive research, the cause of sarcoidosis remains unknown. However, substantial evidence supports the hypothesis that sarcoidosis stems from interactions among genetic and environmental factors, which would account for the significant heterogeneity in this disease across different ethnic groups.

Most patients with sarcoidosis experience spontaneous remission or nonprogressing disease; however, as many as one third of patients develop chronic progressive disease [1]. Clinicians frequently find it difficult to manage sarcoidosis patients due to the significant variability in disease manifestation, diverse organ involvement and multiple non-specific symptoms. Furthermore, outcome measures (e.g., pulmonary function indices, histopathologic abnormalities and high-resolution computed tomography findings) are often nonspecific or have limited prognostic value [2-5].

Over the past decade, the 6 minute walk test (6MWT) has become a popular tool to predict the prognoses of patients with various pulmonary and non-pulmonary diseases, including idiopathic pulmonary fibrosis (IPF), chronic obstructive lung disease, pulmonary hypertension (PH), and chronic heart failure [6-9]. Among patients with IPF, the 6MWT parameters of desaturation [6] and distance [10] can discriminate between survivors and non-survivors. Interestingly, the product of the distance walked during the 6MWT (6MWD) and oxygen saturation (SpO_2_) (i.e., the distance-saturation product, or DSP) is a more reliable indicator of prognosis than either parameter alone [11]. Because the 6MWT is simple, inexpensive, reproducible and well-received by patients (as it mimics the effort required for daily physical activity), it may be a useful tool to track the progress of patients with sarcoidosis in an outpatient setting. Several studies have used the 6MWT to evaluate patients with sarcoidosis [12-15] and have found that the majority of patients exhibit exercise intolerance, which manifests as reduced walking distance. Recently, we reported that several factors are associated with shorter 6MWDs, including gender, percentage of predicted forced vital capacity (FVC), forced expiratory volume in 1 second (FEV_1_), final Borg score and oxygen saturation at the end of the 6MWT [15]. However, that study included only a small number of patients from a single center, which precluded the use of additional tests to predict variables affecting the 6MWT parameters. Moreover, no previous study has examined the relationship between chest CT patterns and 6MWT parameters in patients with pulmonary sarcoidosis.

In the present retrospective study, we therefore sought to determine the relationship between outcome measures of the 6MWT (i.e., distance and the DSP), physiologic parameters and CT patterns in a larger sample of patients from three tertiary hospitals. Furthermore, we tested the hypothesis that the DSP is more effective than 6MWD for identifying variables involved in overall functional status in patients with pulmonary sarcoidosis.

## Methods

### Study Population

Our study population consisted of 59 patients who were diagnosed with pulmonary sarcoidosis, based on biopsy results, between January 2002 and December 2008. Medical records of all patients attended an outpatient pulmonary clinic at one of three study centers, specifically, King Khalid University Hospital, King Abdulaziz Medical City, and Riyadh Military Hospital, in Riyadh, Saudi Arabia were reviewed. The study protocol was approved by the Ethics Committee of each hospital and informed patients consent was waived. Some of the participants in the current investigation were included in our previous studies of Arab patients diagnosed with pulmonary sarcoidosis [15,16]. Sarcoidosis was diagnosed based on the most recent criteria published by the American Thoracic Society (ATS), the European Respiratory Society (ERS) and the World Association of Sarcoidosis and Other Granulomatous Disorders (WASOG) [17]. Data included patient demographics, treatment, pulmonary function, 6MWT, CT and echocardiography findings. The CT reports were collected from medical records and were examined for the following recognized patterns [18]: (1) ground-glass opacity; (2) linear opacity, including interlobular septal lines and interstitial thickening; and (3) features indicating scarring and fibrosis (grouped together), including traction bronchiectasis, honeycombing, cysts, or volume loss. Treatment regimens consisted of none (no treatment), corticosteroids, corticosteroids plus azathioprine, or corticosteroids plus methotrexate. Treatment decisions were based on individual physician opinions.

### Measurements

FVC, FEV_1 _and total lung capacity (TLC) values (Erich Jaeger Masterscreen PFT, GmbH D-97201Hoechberg, Germany) were expressed as percentages of predicted values according to gender, weight and age. Measurements were performed according to the recommendations of the ATS [19]. The predicted values currently used were those of Quanjer and colleagues [20]. Arterial blood gas values (Rapid lab 865; Bayer, Plymouth, UK) were obtained on the same day as the 6MWT was performed. Sampling included partial pressure of oxygen (PaO_2_), partial pressure of carbon dioxide (PaCO_2_) and oxygen saturation (SaO_2_) measurements.

The 6MWT was conducted in accordance with ATS guidelines [21]. All patients exhibited resting oxygen saturation (SpO_2_) > 88% at the beginning of the walk test. Heart rate, blood pressure, oxygen saturation and Borg dyspnea index [22] were recorded at the beginning and end of the 6 minute walk. The total distance walked (in meters) was documented at the end of the test.

The DSP was defined by Lettieri and colleagues [11] as the product of the 6MWD and the lowest room air SpO_2_. For example, if the total distance is 300 m and the lowest SpO_2 _is 88% at the end of the 6MWT, the DSP value would be 264 m% (i.e., 300 × 0.88).

Pulmonary hypertension (PH) was diagnosed based on Doppler echocardiography. Specifically, PH was defined as an estimated right ventricular systolic pressure of > 40 mm Hg in the absence of significant left heart dysfunction, according to criteria established by a World Health Organization symposium on primary PH [23].

### Statistical Analysis

Descriptive statistics [i.e., means, standard deviations, percentages, and 95% confidence interval (CI)] were applied to summarize the continuous and categorical variables. The relationship between two continuous variables was determined by measuring the Pearson's correlation coefficient. Student's t-test for independent samples was used to compare the mean values of continuous variables. The chi-square test was applied to compare the proportion of categorical study variables with that of categorical outcome variables. Backward elimination stepwise multiple regression model for distance and DSP was developed using variables found to be significant (p < 0.05) in the univariate analysis. Statistical Software Package for Social Sciences (SPSS version 13.0; SPSS, Inc; Chicago, Illinois, USA), and PASW version 18.0 were used to analyze the data.

## Results

Among the 59 patients with sarcoidosis analyzed in the present study, 42 (71.2%) were female. The mean age at diagnosis was 47.5 years (range: 19 years to 83 years).

Aberrations in FVC results (< 80% of predicted values) were observed in 30 patients (50.8%). The mean ± standard deviation (SD) for FVC, FEV_1 _and TLC results, as percentages of predicted values, were 77.6 ± 22.2, 77.1 ± 22.8 and 78.7 ± 16.1, respectively. The 6MWT results are summarized in Table 1. The mean 6MWD for the entire cohort was 349 m + 72 m (range: 163 m to 500 m). Thirteen patients (22.0%) achieved 6MWDs > 400 m, 34 patients (57.6%) walked 300 m to 400 m and 12 patients (20.3%) walked < 300 m. On average, females walked a significantly shorter distance than males. The differences are summarized in Table 2 and Table 3. The correlation between factors associated with 6MWD and DSP are shown in Table 4 and Table 5, according to simple regression analysis. 6MWD and DSP were positively correlated with absolute values of FVC, FEV_1 _and TLC. However, DSP was more significantly correlated with these values.

**Table 1:** Outcome of six minutes walk test

Variables	Mean (SD)
Initial heart rate, b/m	93.0 (15.6)
Final heart rate, b/m	109.2 (19.2)
Initial Borg score	0.35 (0.7)
Final Borg score	2.4 (2.1)
Initial SpO_2_, %	95.5 (2.4)
Lowest SpO_2_, %	91.4 (7.3)
6MWD, m	349.0 (72)
DSP, m%	320.0 (75)

SD: standard deviation; b/m: beats per minute;

SpO_2_: oxygen saturation by pulse oximetry;

6MWD: 6-min walk distance; DSP: distance saturation product

**Table 2:** Univariate analysis: Comparison of mean value of distance with categorical variables

Variables	Distance Mean (SD)	t-value	p-value	95% CI
Gender				
Female	324.1 (65.6)	5.0	< 0.0001	513, 119.2
Male	409.4 (44.4)			
				
Pulmonary hypertension				
Yes	313.2 (63.1)	1.85	0.07	-3.9, 90.3
No	356.5 (71.6)			
				
Treatment				
Yes	343.9 (70.9)	1.27	0.21	-16.7, 75.1
No	373.1 (71.8)			
				
Scarring and fibrosis				
Yes	333.7 (68.8)	1.89	0.06	-2.7, 83.9
No	374.3 (71.0)			

**Table 3:** Univariate analysis: Comparison of mean value of DSP with categorical variables

Variables	Distance Mean (SD)	t-value	p-value	95% CI
Gender				
Female	300.8 (70.6)	3.46	< 0.001	28.2, 105.3
Male	367.6 (62.8)			
				
Pulmonary hypertension				
Yes	268.03 (66.1)	2.55	0.01	12.7, 108.3
No	328.5 (72.1)			
				
Treatment				
Yes	311.3 (72.8)	2.04	0.04	0.83, 94.6
No	359.0 (71.9)			
				
Scarring and fibrosis				
Yes	297.9 (70.2)	2.42	0.02	8.8, 98.0
No	3513 (74.2)			

**Table 4:** Simple regression analysis: Relationship between distance and quantitative study variables

Outcome Variables	Independen Variables t	Unstandarized Coefficient		Standarized Coefficient Beta	t-value	p-value	R^2^
		B	S. Error				
DISTANCE	FEV_1_, L	43.81	9.44	0.52	4.64	< 0.0001	0.271
	FVC, L	37.58	8.29	0.51	4.53	< 0.0001	0.262
	TLC, L	25.32	8.26	0.39	3.1	0.003	0.158
	Final Borg score	-10.56	4.62	-0.35	-2.3	0.028	0.121
	PaO_2 _mmHg	2.32	0.79	0.39	2.92	0.005	0.154
	Lowest SpO_2_, %	1.85	1.26	0.19	1.47	0.15	0.036

FEV_1_: Forced expiratory volume in 1 second, FVC: Forced vital capacity;

TLC: Total lung capacity; PaO_2_: Partial pressure or oxygen; SpO_2_: oxygen saturation by pulse oximetry.

**Table 5:** Simple regression analysis: Relationship between DSP and quantitative variables

Outcome Variables	Independen Variables t	Unstandarized Coefficient		Standarized Coefficient Beta	t-value	p-value	R^2^
		B	S. Error				
DSP	FEV_1_, L	52.1	9.3	0.59	5.61	< 0.0001	0.352
	FVC, L	44.8	8.1	0.58	5.49	< 0.0001	0.342
	TLC, L	31.2	8.0	0.48	3.90	< 0.0001	0.233
	Final Borg score	-14.8	4.7	-0.45	-3.12	0.003	0.205
	PaO_2 _mmHg	2.87	0.82	0.45	3.49	0.001	0.206

The CT findings revealed ground-glass opacity in 32.2% of the patients, linear opacity in 27.1% of the patients and scarring and fibrosis in 40.6% of the patients. Comparison of patients with and without ground glass opacity revealed a shorter walking distance for the former; however, no significant differences were observed across groups [DSP (m%): 299.2 + 71.8 and 399.3 + 76.0, respectively; *p *= 0.08; distance (m): 337 + 72.7 and 363 + 70.7, respectively; *p *= 0.24]. No statistical differences in 6MWD were noted between patients with and without linear opacity [DSP (m%): 301.5 + 68.4 and 332.3 + 78.8, respectively; *p *= 0.21; distance (m): 331.8 + 67.8 and 362.2 + 73.0, respectively; *p *= 0.19]. A significant difference was noted for DSP, but not for 6MWD, when patients with lung fibrosis were compared with those without fibrosis. The differences are summarized in Table 2 and Table 3.

Doppler echocardiography was performed in 46 patients (78%). Of those patients, pulmonary artery systolic pressure > 40 mm Hg was documented in 12 patients (23%). Differences in mean DSP values were greater than those in 6MWD when patients with PH were compared with those without PH. The differences are summarized in Table 2 and Table 3.

We assessed the effects of treatment on the 6MWD and found that 47 patients (79.6%) were administered corticosteroids alone or in combination with immunosuppressive therapy, while 12 patients (20.3%) did not receive any therapy. Patients receiving therapy achieved shorter 6MWDs than those not receiving treatment. In these cases, a significant difference was noted for DSP, but not distance shown in Table 2 and Table 3.

Significant variables identified by univariate analysis were assessed in a stepwise multiple regression model, using backward elimination method. Separate models were developed for distance and for DSP. The models included gender, FEV_1_, FVC, TLC, final Borg score, lowest SpO_2 _(distance model only), PaO_2_, presence of CT fibrosis, treatment status and presence of PH. In the distance model, gender and absolute value of FEV_1 _were identified as significant independent variables. Whereas, in the DSP model FEV_1 _was more strongly related. Results are shown in Table 6, and Table 7.

**Table 6:** Stepwise regression analysis (backward elimination method): Relationship between distance and study (categorical and quantitative) variables

Outcome Variables	Independen Variables t	Unstandarized Coefficient		Standarized Coefficient Beta	t-value	p-value	95%CI	R^2^
		B	S. Error					
DISTANCE	Gender	-43.5	16.7	-0.35	-2.56	0.016	-78.3, -8.7	0.45
	FEV_1_	46.1	11.7	0.54	3.95	< 0.0001	22.2, 69.9	

**Table 7:** Stepwise regression analysis (backward elimination method): Relationship between DSP and study (categorical and quantitative) variables

Outcome Variables	Indepndent Variables	Unstanardized Coefficients		Standardized Coefficients Beta	t -value	p-value	95% CI	R^2^
		B	S. Error					
DSP	FEV_1_	43.4	17.3	0.489	2.5	0.02	6.4, 80.4	0.53

## Discussion

Our results demonstrate that exercise intolerance among patients with pulmonary sarcoidosis manifests as shorter distances walked during the 6MWT. We have identified several factors that contribute to reductions in 6MWD, including gender, pulmonary function parameters, dyspnea score, and PaO_2_. Furthermore, DSP was more significantly correlated with physiologic indices than was 6MWD. Our findings demonstrated that DSP, but not distance, was significantly associated with CT fibrosis, systemic therapy and PH.

Traditionally, pulmonary function tests (PFTs) have been used in the management of individuals with pulmonary sarcoidosis to identify patients with pulmonary involvement and to follow the course of the disease. However, the predictive power of these tests is limited [24,25]. Furthermore, the correlation of dyspnea with PFTs has produced discordant results among patients with sarcoidosis [26,27], suggesting that additional methods for the evaluation of functional status are needed. Consistent with previous studies, our data demonstrate that FVC, FEV_1 _and TLC are significantly correlated with 6MWD [12,15]. However, DSP showed a much higher correlation with pulmonary function parameters than did 6MWD alone.

Previous studies that used the Borg score to measure the perception of breathlessness during exertion have yielded inconsistent results. Mador and colleagues [28] found that Borg ratings of dyspnea among patients with chronic obstructive pulmonary disease were highly variable at submaximal levels of exercise compared with maximal levels of exercise, whereas Mack and associates [29] noted a better correlation when patients performed a walking test rather than a maximal exercise test. We found a significant inverse correlation between Borg score and 6MWD, consistent with previous studies [12,13,15]. Interestingly, a stronger correlation was noted for DSP than for distance. Although the lungs are the most commonly affected tissues in patients with sarcoidosis, exercise intolerance can also be attributed to other factors such as cardiac involvement and skeletal muscle weakness.

The presence of PH was recently identified as an important factor affecting the survival of patients with sarcoidosis [24,30,31]. In the present study, 12 patients with PH were identified. We observed a reduction in walking distance among the patients with PH, consistent with previous reports [12,32]. A statistically significant difference was noted for DSP, but not distance, when patients with PH were compared with those without PH. Many factors (e.g., age, height, weight and effort) may have major effects on 6MWD, which may explain why we did not identify any statistically significant differences in walking distance when we compared patients with and without PH. Bourbonnais and Samavati [32] reported that the level of oxygen saturation at 6 minutes was better than walking distance at predicting the presence of PH in patients with sarcoidosis. However, the purpose of that study was to identify variables that could predict the presence of sarcoidosis-associated PH, whereas the present study was designed to correlate 6MWT outcome measures (i.e., distance and DSP) with various physiologic parameters, including the presence of PH. Additional large-scale studies are warranted to explore the value of DSP as a screening tool to identify PH in patients with sarcoidosis.

Skeletal muscle weakness has a significant impact on exercise tolerance in patients with sarcoidosis [14]. Factors that play a role in the development of muscle weakness include myopathy, corticosteroid therapy, fatigue and physical deconditioning. Patients receiving corticosteroid therapy in our study achieved shorter 6MWDs than did those not receiving such therapy; interestingly, this difference was significant for DSP but not 6MWD. Although Baughman and Colleagues [12] noted significant differences in 6MWD between patients who took corticosteroids and those who did not, Spruit and associates [14] found no difference in muscle force or 6MWD between patients taking corticosteroids and those not receiving such treatment. This discrepancy may reflect the marked heterogeneity in disease progression, duration of corticosteroid use, effects associated with race and ethnicity, as well as extrapulmonary organ involvement, fatigue, or physical deconditioning. Thus, future studies are needed to explore the possible causes of skeletal muscle weakness among patients with sarcoidosis.

While CT and high-resolution CT play a pivotal diagnostic role in the evaluation of diffuse interstitial lung disease, including sarcoidosis, routine CT scanning is not advocated in the management of sarcoidosis, except in patients with normal or atypical chest radiography or specific complications such pulmonary fibrosis, bronchiectasis, aspergilloma or malignancy [17,33]. Several studies have correlated CT patterns with pulmonary function tests [2-4] and CT findings with pathological abnormalities [5,34,35] among patients with pulmonary sarcoidosis. However, CT patterns have never before been correlated with 6MWT results. In the present study, 40.6% of the patients exhibited CT patterns indicative of lung fibrosis. Although patients with lung fibrosis tended to walk shorter distances, the difference in 6MWD between groups was not statistically significant. By contrast, the DSP value did differ significantly between patients with and without lung fibrosis, suggesting that DSP has the potential to identify a subset of patients with pulmonary fibrosis and therefore may provide indirect information about global functional status. Discordance in the outcomes of patients with ground glass attenuation has been observed in several studies [3,4,36]. Furthermore, attempts to correlate ground glass patterns with pathological findings have led to variable reports of granulomatous infiltration or fibrosis [34,35]. We noted a trend toward statistical significance when DSP was used to assess patients with and without ground glass opacity. Future studies should use DSP to correlate the reversibility of ground glass opacity with 6MWT results in response to therapy.

The present study found that female gender was associated with a significant reduction in 6MWD. This effect persists when multiple regression analysis was applied for the distance but not for DSP. Furthermore, we identified FEV_1 _as predictive factor among our cohort. Our results conflict with a previous report by Baughman and colleagues [12], who found that FVC, oxygen saturation with exercise and self-reported respiratory health were independent variables associated with 6MWD.

Exercise among patients with sarcoidosis requires a global and integrated response of the cardiovascular, respiratory, neuromuscular and metabolic systems, which might explain why FEV_1 _accounted for 53% of the changes in DSP and 45% of the changes in 6MWD. These results indicate that factors other than lung function may have a significant effect on 6MWD. Baughman and colleagues [12] found that activity scores on the St. George Respiratory Questionnaire were more effective than FVC at predicting 6MWD results. In another study, Kabitz and colleagues [13] found that inspiratory muscle strength was more effective than FVC or resting PaO_2 _at predicting 6MWD results. However, neither of these studies determined the predictor variable that accounts for total variance (i.e., r^2^) for 6MWD, thus precluding meaningful comparison with our data.

Both 6MWD and desaturation during 6MWT are valuable tools for predicting functional capacity and mortality in various pulmonary and non-pulmonary diseases [6-9]. Thus, use of the outcome measure, DSP, in conjunction with 6MWT and other tests may help with the management of patients with sarcoidosis by providing additional information about functional status. In addition, DSP may serve as a useful assessment tool for monitoring patient responses to therapy.

The present study had several limitations. First, our work is a retrospective review wherein clinical information was obtained from hospital records. Our clinical setting is a tertiary-care hospital where selection bias toward patients with more advanced disease is inevitable. Finally, small number of patients in our cohort relative to the studied variables mandates external validation of our prediction model before it can be used in clinical practice.

## Conclusions

Several factors were associated with a reduction in 6MWD among patients with sarcoidosis. When DSP was used instead of 6MWD, we identified several additional factors associated with reductions in 6MWT performance, including CT fibrosis, systemic therapy and PH. Stepwise multiple regression analysis revealed that FEV_1 _was an independent predictor of 6MWD and was a significantly better predictor of DSP than of 6MWD. This study is the first to demonstrate a correlation between 6MWT results and CT fibrosis, and suggests that DSP is a potential indicator of functional status in patients with sarcoidosis.

## Competing interests

The authors declare that they have no competing interests.

## Authors' contributions

The principle author (EHA) prepared the study proposal and wrote the manuscript. SAS reviewed the data and performed the statistical analysis using appropriate statistical methods. MMI and MOA helped interpret the data and assisted with writing the manuscript. ACI reviewed and helped interpret the data, in addition to assisting with the preparation of the written manuscript. All authors read and approved the final manuscript.

## Pre-publication history

The pre-publication history for this paper can be accessed here:

http://www.biomedcentral.com/1471-2466/10/42/prepub
